# Principles of Dental Surgery; Exhibiting a New Method of Treating Diseases of the Teeth and Gums, &c.

**Published:** 1827-01

**Authors:** 


*Principles of Dental Surgery; exhibiting a New Method of Treating the Diseases of the Teeth and Gums; especially calculated to promote their Health and Beauty, accompanied by a General View of the Present State of Dental Surgery, with occasional References to the more prevalent Abuses of the Art: in Two Parts.* By Leonard Koecker, Surgeon-Dentist, Doctor in Medicine and Surgery, &c. &c. 8vo. pp. xxii. 445, London, 1826.

Mr. Koecker has divided his Work into two parts; and, as the arrangement is convenient, it shall be adopted in the present article, wherein we propose to consider *generally* this author's "doctrines," and to exhibit *particularly* a concise view of the principles which ought to guide the surgeon-dentist in his treatment of disease in the teeth.

PART I. Chapter I. *General Observations on the Present State of Dental Surgery, and the Development of the Author's Method of Treatment.* In the seventeen pages he assigns to this section, Mr. K. avers, that the doers of "dentistry" in Germany, France, America, and England, are bungling, unskilful, and injudicious operators; that the abuses of dental surgery are "quite the order of the day," and by no means confined to the practice of the lower orders of *our body*, but their effects are very frequently to be observed in that of dentists generally considered of the first rank, highly esteemed by the public, and held of good reputation even by the medical profession; that there is no branch of the healing art so little founded on scientific principles as the dental surgery, this having been so long in the hands of illiterate men whose names, he says, are loaded with high-sounding titles; that empirics are the natural offspring of the imperfection and novelty of a science, but the proper and most effectual remedy against empiricism, is an improved knowledge of the principles of the dental art, as a branch of surgery; and that the author does not hesitate in the least to assert it as his sincere opinion, that dental surgery, in the manner in which it is practised at present, is really a great positive evil. These comprehensive statements are illustrated by five cases which Mr. K. we doubt not, regards as being very instructive as well as impressive; for, he declares, their publication arose entirely from his great anxiety to promote the substantial interests of the profession and to put a stop, if possible, to the pernicious doings whereby it is so lamentably disgraced. Most willing, however, as we are to regard Mr. Koecker's manner as well as his matter with indulgence, it is every way requisite that, in admitting his having shewn this much, we require to be understood as having a perfect recollection of the relations which exist between unmeasured assertion and the kind of evidence that leads to inductive demonstration.

Each of the learned professions—we feel humiliated by the admission—has ever been pestered by the intrusions and mischievous absurdities of quackery; but there would be great unreasonableness, and manifest injustice as well, in concluding, from this circumstance, that most, or even many, of its members should be considered as rude and Charlatanical pretenders. Entertaining this view of the case, therefore, and considering the "professors of dental surgery" fairly entitled to the benefit of being appreciated every one by his own merits, we cannot pass from reminding Mr. K. that his "reprobation" of his "professional brethren" is too indiscriminate, and his mode of determining an estimate of their art rather uncharitable and precipitate. Without adverting, on this occasion, to the systematic or elementary works on anatomy, physiology, and pathology, which necessarily include the subjects of dentition in most of their healthy and morbid relations, we might direct his attention to a multitude of monographs on the natural history, functions, and diseases of the teeth; many of which, besides exhibiting the clearest proofs of their authors' *mechanical* excellence, are distinguished, in a pre-eminent degree, by the philosophy of scientific observation and research. Our advice to Mr. K. therefore, and our request of our readers is, that they examine, among the rest, the writings of Strumard,*(a)* Hoffman,*(b)* Fauchard,*(c)* Lavani,*(d)* Schaeffer,*(e)* Jourdain,*(f)* Hunter,*(g)* Campani,*(h)* Blake,*(i)* Broussonnet,*(k)* Fox,*(l)* Tenon,*(m)* Bœtigger,*(n)* Duval,*(o)* Lavagna,*(p)* Gallette,*(q)* Laveille,*(r)* Miel,*(s)* and Serre,*(t)** and compare impartially with theirs the number and value of Mr. Koecker's discoveries and improvements in dental surgery.

"In publishing this manual, the author has been *partly* influenced by the desire of proving that he is not unworthy of the confidence with which he has been honoured."—"The principles on which he has for many years practised this art, being essentially different from any hitherto known, and considering those which are commonly received as being founded on superficial views and pernicious errors, he has deemed it useful to give a detail of his peculiar ideas in such a manner as, he trusts, will not only be found to deserve some attention on the part of the professional reader, but also at the same time, calculated to prepare the public mind at large for their reception." P. vii.

Animadversion is not the sort of thing whereof we are particularly enamoured; nevertheless, since Mr. K. has chosen throughout his volume to be so very severe on others, he cannot object to our taking occasion, here and once for all, to remind him, that even the most generous examiner might find just reason to accuse him of what might be considered too indiscriminate censure. This much we feel ourselves obliged by every duty to say; but at the same time we add, with infinitely greater satisfaction, that Mr. K.'s book comprises much instructive matter, which we hope, one day, to see divested of all its cumbersome and offensive accompaniments. Since, however, by the advice of his friends, he has ventured to make public the "result of his experience" "for the advancement of the profession and the best interests of humanity," we shall, out of gratitude for his good intentions, inform him that from the "*medical* profession," he may be assured of the most compassionate indulgence; but that, from the "professors of dentistry," if they think of paying him in kind, he may expect more hard words than kindliness and candour: 'tis a good rule in all manner of speculation, especially in authorship—*veniam quam petimus dabimusque vicissim.*

CHAP. II. *General Principles of Dental Surgery.*—Mr. K. regards this as the "*science*" of preventing and curing the diseases, and of preserving the health and beauty of the teeth and of the parts connected with them: it includes also the "remedial treatment" of defects, or loss of parts injurious to health, general appearance, and to the power of distinct articula-

* See notes at the end of this article. They were too long to be inserted in this place.—*Ed.*

tion, and, consequently, the practice of this "very useful *art*" should be established on a solid foundation. To obtain these important objects, he says it is indispensably necessary to have a minute and comprehensive knowledge of—the natural history of the teeth and gums in their healthy state—the diseases to which they are subject, the symptoms of these, and their remote as well as proximate causes, at the successive stages of their growth—the connexions, sympathies, and influences of the teeth, gums, sockets, periosteum and the maxillary bones, generally and individually, upon each other, in their sound and morbid states; and especially those of the first set upon the permanent teeth—the effects produced on the teeth and contiguous parts by disease of particular structures and disorder of the constitution, at different periods of their development—the medical and surgical remedies, and their proper application in treating unhealthy teeth —and the surgical and mechanical means required by the surgeon-dentist, with a general knowledge of the collateral mechanical arts.

This arrangement of "Principles" has the merit of being very distinct as well as judicious: it is followed, moreover, in the remaining six chapters of this part, with many sensible, although *general* remarks on each of these principles, in the form of explication and doctrine; but, as the author has entirely omitted all reference to the sources from which the *particular* philosophy of his favourite "science" can be drawn, our readers will perceive the value of the select bibliographical list which this article contributes to their service. Mr. Samuel Cooper would essentially promote the "advancement of his profession in connexion with the best interests of humanity," were he to introduce, into his most useful Dictionary, a section on the organization and diseases of the teeth: an article on these subjects would increase the value of that excellent work which, decidedly above all others, gives the best and most comprehensive view of the principles and practice of surgery.

Chap. III. *General Remarks on the Natural History of the Teeth and their Relative Parts, with Pathological Observations.*—Our author confines himself, in this chapter, to such observations as are immediately subservient to his present inquiry, and refers the reader to the works of Hunter, Blake, and Fox, for a more extended examination of this "important study." At p. 37 he remarks—

"From the rudiments of the temporary teeth those of the permanent are formed: at an early period of their formation both are firmly and closely attached to one another, and contained in the same socket; but, as they advance in their growth, the sockets become enlarged, a separation of the pulps and a division of the sockets take place, and a separate socket is formed for both: in this state they remain intimately connected by the means of their membrane and the gums. From a due consideration of this fact, the great sympathy and influence between the first and second set of teeth become perfectly evident; a fact requiring a very comprehensive consideration in the treatment of children on the shedding of their teeth."

Mr. K. is of opinion that the teeth, notwithstanding they consist of the hardest and most solid bony texture, are nevertheless organized, vascular, and interwoven with nervous and arterial fibres. We do not dispute the accuracy of this doctrine, but we can admit it only as matter of analogical inference: every thing that grows must have life and be organized; consequently the teeth are vital and composed of organic structure; if they were protected from the external air, and the influences of chemical and mechanical agents, and, when diseased, were cleaned and dressed as bones are, it might be *inferred* that they would regenerate their structure as other organs do. Mr. K. has *said*, but not *shewn*, that the teeth are *vascular*; but it is a fundamental rule in philosophy, never to assert what cannot be demonstrated: his pathological observations are concise, but valuable.

CHAP. IV. *General Remarks on the Diseases of the Teeth and their relative Parts.*—Nature has destined the teeth to live and die only with the general constitution: Mr. K. infers this doctrine from the fact, that such is the case with the brute animals in which the teeth are not exposed to the same morbific causes that occasion the loss and destruction of these organs in man. We quote what follows as legitimate and ingenious reasoning:—

"If we see, for instance, (p. 45,) an individual after the age of thirty years, in a most delicate state of health, with a beautiful and perfect set of teeth, we may safely infer from this fact, and we will generally find it so, that Nature had originally given him a good constitution, and that, at an early period of life, he was not suffering from any disease of consequence; but, that some later causes or accidents must have impaired his naturally good constitution. If, again, we observe an individual, of a very robust constitution, possessed of a very diseased set of teeth, we may also frequently find that, at an early period of life, he must have been the subject of constitutional disorder, to the influence of which should be ascribed the actual imperfections or diseased state of his teeth. In these instances, however, much greater circumspection is requisite than in the former, inasmuch as no positive conclusion could be drawn, and no prognosis should be made, before we have positively ascertained the actual fact, that the diseased state of the mouth is not the effect of some mechanical or chemical cause, such as accidental violence, injudicious operations, or other mal-practices, of incompetent dentists."

No doubt can be entertained of the general accuracy of these positions: they are exceedingly important, and ought never to be lost sight of in practice. Mr. K. thinks the diseases of the teeth and parts connected with them are not many, and the judicious management of them is not rendered difficult so much by their number as by their great varieties of situation, character, and stages, and a multitude of other perplexing circumstances, which obstinately resist their curative as well as preventive treatment.

CHAP. V. *General View of the Causes of the Diseases of the Teeth and their relative Parts, with their Mode of Action.*—These causes are either general or lo[illegible]: under the first head are classed constitutional diseases, constitutional changes, diet, climate, and medicine; under the second, diseases of the mouth, tartar, saliva, and external applications. All constitutional maladies, and such local diseases of important organs as affect the whole system, and, through it, individual parts, must be presumed, Mr. K. says, to extend their morbid influences to the teeth as well as gums: it also appears that, in consequence of the intimate sympathy subsisting between the teeth—supplied as they are with nerves of extreme sensibility, and with arteries at least not less irritable than arteries in other parts of the body—and the general system, that the diseases of the latter must be productive of more extensive effects upon the former than upon other parts generally, especially when affected by local disease: this fact, he declares, has been proved to him beyond any doubt, by repeated observations and long experience. In chronic diseases, the teeth generally assume a pale or milky-bluish appearance, and become more or less "opaque;" while, in acute fever, they acquire an "inclination" to a reddish hue. There is some originality and much instruction in this quotation:—

"After death from violent fever, (p. 51,) I have found the blood in the blood-vessels of the lining membrane of the teeth sometimes very dark, so that the external appearance of the teeth was quite red: and, at other times, great parts of the bony structure had also *turned considerably red, from the great injection of the blood, and from its coagulation in the minute vessels of these parts;* this, by maceration during two or three months, I could not succeed wholly to extract."—"The gum-boil is a local disease produced by mortification in the socket, and suppuration and inflammation in the periosteum and the gum, originally and proximately occasioned by dead roots or teeth; and, though very small in its external appearance, is an affection of no little importance, it being constantly progressive in its nature, and totally destructive in its event: its real exciting cause is much more frequently indigestion than the action of cold."—"In persons having calculous affections, there is a remarkable tendency to deposition of tartar on the teeth, and it is always necessary to use powerful means to resist its re-accumulation after being perfectly removed."—"The prevalent idea that consumptive persons have good teeth, because they are often delicately white, is altogether groundless: these subjects are generally young people, and such as have not lost their front teeth; and the diseased action, produced in them by the constitutional disorder, gives to them the opaque, pale, or bluish appearance, which is mistaken, by the inexperienced eye, for a symptom of health, while, in reality, it is an indication of a morbid state of the lining membrane; in fact, so far from finding good teeth in such persons, I have generally found them comparatively defective and diseased."

Mr. Koecker's remarks on constitutional changes are very limited, being chiefly confined to pregnancy: those on diet and climate are concise also: what he says on medicine will be found accurate and interesting: he finds himself authorised in considering all medicines, properly administered, to be as beneficial to the teeth as they are to the other parts of the animal system: he would not even except the most powerful preparations of mercury, arsenic, or the acids, when skilfully exhibited; but he thinks there is neither rashness nor uncharitableness in attributing a large proportion of the local and general diseases of the system, and consequently of the teeth, to the abuse of medicine.

Passing, with a short notice, the diseases of the mouth, the tartar and saliva, as *local* causes of disease in the teeth and gums, Mr. K. enlarges on the misapplication of external remedies; among which he ranks tooth-powders, tooth-pastes, tinctures, essential oils, and mixtures of all kinds; tooth-brushes and tooth-picks. Of all external causes, however, none are more active and mischievous than the unskilful operations of cleansing, scaling, filing, cutting, plugging, and extracting the teeth: but, he adds, "the most cruel and destructive of all morbific causes known in dental surgery, are those recommended by Hunter, Fox, and others, namely, destroying the nerves of the teeth, replacing a tooth in its socket after its previous extraction, transplanting teeth from the mouth of one person into the mouth of another, applying the actual cautery for arresting hæmorrhages from the socket after the extraction of a tooth, using ligatures for retaining in their places artificial teeth, or such diseased teeth as are loose, or rather the sockets of which are destroyed by disease, and inserting artificial teeth without the necessary surgical principles, and great judgment and skill." These are the *ipsissima verba* of Mr. Koecker, and we do think they call for serious consideration.

Chap. VI. *On the Morbid Effects of the Diseases of the Teeth and Gums*

*on the General System.*—This section contains many instructive observations: we submit a fair specimen in the shape of an extract.

Diseases of the teeth "act like slow poisons, slow but sure: they will destroy either in a direct or indirect way; but towards destruction they certainly tend. In strong constitutions, which are little influenced by local diseases of the mouth, and in which the affected parts are not excited by general causes, or irritated by previous mal-treatment, the maladies of the teeth, and still more those of the neighbouring parts, may proceed to a considerable extent, without being accompanied by very painful symptoms, either local or general, and even without much apparent inconvenience. In such instances, the diseases of the mouth are so slow and gradual, yet so certainly progressive in their chronic state, that sometimes not one part connected with the teeth is left in a perfectly sound state; until at length the whole catalogue of the diseases of those parts meet in one and the same case: and, in the same regularly slow and progressive manner in which these maladies proceeded, they will also again decrease, and Nature alone will effect a perfect cure by the removal of every tooth, and by the consequent annihilation and total absorption of the alveolar processes. The general health, in cases of this kind, always maintains its predominance over local disease, and one diseased part becomes the means of effecting the destruction of another. The principal local causes of the diseases of the gums, periosteum, and alveoli, being decayed or dead teeth and tartar, and the principal causes of the maladies of the teeth themselves being the diseases of the gums and sockets, the diseases of the gums are cured by Nature only, by the removal of all the teeth and tartar, and by absorption of the alveoli and gums. This progress of disease in the teeth and gums is most frequently observed in the more healthy and ignorant classes of society, whose teeth are generally left to Nature, in consequence of which they frequently suffer from neglect, but very seldom from artificial causes; for the nervous sensibility is, generally, much less in uneducated people, and the constitution less irritable than in the enlightened and polite classes of society."—"The important connexion between the teeth and gums on the one hand, and the general system on the other, and the exceedingly great influence, whether favourable or otherwise, of the former upon the latter, have not hitherto received so much attention on the part of physicians and surgeons as they really deserve. Would the more liberal members of the healing art determine their earnest attention to the diseases of the teeth as causes of disease in the general system, I feel confident that it would contribute, in no small degree, to the extension of their professional powers. In those affections which are generally classed in the list of nervous and rheumatic diseases, whether chronic or acute, the teeth, if in any way disordered, may always be suspected to be the principal causes. The immediate connexion of the teeth and other parts of the mouth with the digestive organs and the lungs, renders it still more probable that the maladies of the teeth may very frequently constitute a very considerable part of the exciting causes of primary and secondary derangements of these important parts; while, it is also very evident that, in consequence of their close relationship with the brain, the delicate organs of the senses of hearing and of sight must very often be much impaired and painfully affected during diseased states of the teeth, gums, alveoli, maxillary bones, and their investing membrane."

Diseases of the teeth, according to Mr. K's observations, are much more common in America than any other country: they are also much more acute and violent in their symptoms, and by far more rapid in their progress than perhaps in any other climate.

Chap. VII. *Treatment of the Diseases of the Teeth and their relative Parts.* This chapter is subdivided into two sections, entitled, Introductory Observations, and a View of the Author's Principles and Method of Treating the Diseases in Question : under each of these will be found not a few important remarks, occasionally embarrassed, however, with repetition. Mr. K. informs us, that the chief curative means of the dentist are operations, many of which produce considerable irritation ; and, hence, if unseasonably performed, they aggravate instead of curing the malady, and also extend its injurious effects. Almost every disease requires a different treatment: in complicated cases, therefore, it is necessary to ascertain their several causes ; for, in the removal of these, which is not to be effected without consecutive operations, the success of the treatment depends much on the manner in which such operations are performed ; in practising them it is proper to avoid precipitation as well as delay, as being equally dangerous. Acute diseases of the teeth and mouth should be first considered in the treatment, with reference to the general health and that of the parts; and next, their chronic symptomatic affections, which exercise the same hurtful effects upon the system as on the seat of the malady. Our author's doctrine, in fine, is, that the cure of disease in the teeth and gums depends upon a skilful removal of its morbid causes, and particularly upon the order and time of the operations necessary for this removal. He divides these causes into the acute and chronic, which again are subdivided into general and local.

"All acute diseases of the system," he observes, (p. 134) "whether general or local, act more speedily and destructively upon the teeth and gums than any other causes ; and, of local diseases, those of the mouth in particular. The latter produce the greatest symptomatic inflammation of the different parts of the teeth and gums ; and, as they are also, in most instances, not a little painful, they should be first attended to. The dental treatment, at any period of an acute disease of the general system, accompanied with pain and other acute symptoms in the teeth or their relative parts, should never be more than partial, amounting only to the removal of such causes of the local maladies of the mouth as are presumed to produce immediate pain or acute inflammation ; and this should be done in the most cautious manner. Even when chronic maladies have so much affected the patient as to have confined him to his bed or room, and to have been accompanied by acute inflammation of the gums and teeth, it will be proper to observe the same treatment. But when the disorder is not of that violent kind, the whole system will be greatly benefited, not only by a removal of such local exciting causes as occasion the acute and painful diseases, but also by the additional removal of all presumed causes of chronic maladies of the teeth and gums ; and this treatment should be accompanied by a proper and judicious regard to the state of the general health and constitution. These principles should be particularly observed in respect to females in time of pregnancy or suckling, inasmuch as a cautious treatment of the teeth and gums *will not benefit the health of the mother only, but also that of the child, and even the future soundness of the latter's teeth.*"

These last remarks are exceedingly important, and ought to fix the attention of every obstetrical practitioner. Our author states that, in no instance has he been deterred from performing any operation, of which the necessity was sufficiently indicated, by the circumstance of pregnancy or suckling, provided the person was willing to undergo it ; and particularly in those cases where it was to be instituted, with a view to relieve the patient from immediate suffering. He extracted fifteen decayed and dead teeth and roots, in the space of twenty-five minutes, from the mouth of a lady in the fifth month of pregnancy, with the best results.

Mr. K's account of his principles and method of treating the dental diseases is not very perspicuous: it is illustrated, however, by several minutely detailed cases; and, in one to which he makes particular reference, the patient underwent sixty-two operations, occupying, at different sittings, from one to three hours each: a summary of these will exhibit the progressive stages of his treatment. *First,* nine operations were executed for the extraction of carious teeth and roots; 2*d.* Two for scaling the teeth; 3*d.* Twenty-eight for removing the superficial caries with the file and cutting instruments; 4*th.* Twenty-two for the extirpation of caries, and for stopping the cavities; and 5*th.* One for treatment of the lining membrane after it had become exposed. In our author's general principles, in fine, we see much good sense and sound pathological philosophy; but we could have wished that he had given some plates of his instruments, and a more detailed account of his method of treatment, in order to prevent his enemies accusing him of holding back information which it was in his power to convey.

CHAP. VIII. *Preventive Treatment necessary for the Preservation of the Health and Beauty of the Teeth aud Gums.*—Mr. K. remarks very justly, that to enable the dental surgeon to discover, and then to remove or counteract, the causes which arise from general diseases or local affections of the mouth, a complete general physiological and pathological knowledge is requisite. Although, he says, general diseases have not any immediate and permanent effect upon healthy teeth, still it is known that they injure them indirectly, and sometimes occasion such local maladies of the parts surrounding the teeth as determine, at one time or other, the accession of idiopathic diseases in these parts, and the consequent loss of the teeth themselves. When the gums and teeth, he adds, are in a healthy state, general diseases can be considered only as causes of symptomatic affections, or of future diseases only; but, if the effects or changes they produce are not attended to in proper time, some affection of the teeth, or of the gums and socket, may be the consequence. These are adduced as reasons for advising the attendance of a dentist in consultation with the medical attendant.

After stating generally the causes of local disease in the teeth, he observes, that attention to the removal of these causes is particularly requisite in every case after general disease, confinement in childbed, suckling of children, and voyages at sea. He has never been obliged, he adds, to repeat an instrumental cleaning, after having once restored the teeth to perfect health, except when some particular cause or great neglect of the patient's has rendered the repetition unavoidable; and this leads him to deprecate the custom of having the teeth cleaned by a dentist, regularly every six or twelve months, which, he says, is about as ridiculous and as mischievous as the exploded one of taking a strong dose of medicine once in the month, for the purpose of preserving the digestive organs in a healthy state. The remarks on the means of the preservative dental treatment, which occupy the best half of this chapter, are very general.

CHAP. IX. *Treatment of the Teeth and Gums of Children at the Second Dentition.*—Nothing more than successive applications of "proper preventive means" is necessary for treating the teeth, at this period, in strong and healthy children. These means, according to our author, are *first,* to assist Nature in its natural process, if this is too slow, by removing those temporary teeth, whose roots are too slowly or irregularly absorbed, even though they should be free from disease; *second,* to retard this process, if it is too active, by preserving all the contiguous parts of the remaining first teeth, and of the permanent teeth, which are already visible, in a healthy state; by which means, every unhealthy and irregular action, and consequently too rapid a progress in some, and too slow a progress in others, are prevented;—by the removal, also, of all such temporary teeth as are painful, from the lining membrane being irritated by caries;—and, likewise, by strict attention to keep the mouth perfectly clean. *Third,* to prevent all morbid influence of the temporary teeth upon those of the permanent set,—by employing, as suitable, the means already enjoined, and by extracting such of the temporary as are so diseased that their caries is brought into contact *(does this imply the possibility of contagion?)* with the permanent teeth, particularly with their lateral surface, even though the former should be free from pain. In addition to this local treatment, Mr. K. recommends attention to the peculiarities of constitution, enjoyment of a wholesome atmosphere and a nourishing diet, perfectly free from stimulating properties.

Irregularity of the teeth is regarded by our author as one of the chief predisposing causes of their diseases, and never fails, even in the most healthy constitution, to destroy, sooner or later, the strongest and best set of teeth unless properly tended. These effects of irregularity are made by far too exclusive, we should think; and the unpleasing, grinning, or forbidding appearance of the features and countenance, ascribed to the same source, is not quite universal. He enjoins especial attention to "these disorders" at an early period of life, and to extract some of the permanent set, in order to give sufficient room for the rest. Instead of the incisor, cuspid, and bicuspid teeth, he prefers the first molar for extraction, because these are generally most predisposed to disease, are least important, and so situated as to afford, by timely removal, sufficient space for the anterior teeth, as well as the second and third grinders: the operation should be performed at any period before the age of twelve years; and it is absolutely necessary that all the four molar teeth be extracted at the same time or at very short intervals. With two cases this chapter ends: we have given the substance of it.

PART II.—At which we are now arrived, comprises the history and treatment of the most common diseases of the teeth and gums, with the methods of performing dento-surgical operations.

Chap. I. *Caries of the Teeth.*—This chapter is introduced by some general remarks, and then subdivided into two subordinate sections. With respect to caries, Mr. K. holds it to be the most afflicting and destructive disease to which the teeth are liable, not unfrequently spreading its ravages until every tooth is destroyed: he asserts, also, that this malady has hitherto not been properly understood, nor satisfactorily described by any surgical writer. One great cause of this, he thinks, is the surprising manner of confounding the disease with its effects—"putrefaction;" or, in other words, the mistaking a living tooth under the influence of disease with a dead tooth which disease has destroyed.

"Caries of the teeth," he states, p. 208, "must be considered as similar to gangrene of other parts of the system: and, where we speak of caries as a disease, we mean that diseased action in the bony structure of the living tooth, which is produced by the chemical irritation of its rotten or dead parts. Hence, it is indispensable that we should make a due distinction between caries considered as a disease in the teeth, and the effect of that disease; namely, mortification and putrefaction of its whole structure: a carious tooth, properly so called, is a living tooth possessed of that vital action by which the morbid irritation of the dead substance is counteracted. By this counteraction, more or less inflammation is produced in the bony structure of the tooth; whereas, a tooth deprived of this vital action, is no longer carious but dead. This distinction is of the greatest importance, not only inasmuch as the effects of diseased and dead teeth are very different, but, also, as they almost invariably require an opposite surgical consideration and treatment."—"Caries," p. 211, "in fact, is that state of the teeth in which mortification has taken place in one part, and inflammation in the part contiguous to it, the former originally produced by the latter, and the latter kept up by continual contact with the former. The rapid progress of diseased action must consequently more quickly destroy the living tooth, than the chemical agency by which dead matter acts upon a bone of so hard and dense a structure as a human tooth; and the less, therefore, it is enabled by its very dense construction to arrest or suspend diseased action when living, it is naturally the more adapted to bear the most powerful chemical and mechanical influences, such as putrefaction and mortification, in its dead state. This may be frequently observed in dead roots, which remain after the crumbling away of their crowns; the roots are sometimes particularly protected by the surrounding parts."

We stay not at present to inquire into the accuracy of these pathological distinctions, or to express a desire of knowing the evidence on which they are based: neither have we inclination to review Mr. K's. criticisms on Hunter, Fox, and Duval, which, though perhaps just in a few respects, are undoubtedly expressed with little courtesy. He divides the treatment of caries into three parts;—of the *simple*, or that form of it wherein the bony structure of the tooth is exclusively implicated;—of the *complicated*, or when it is accompanied by disease of the lining membranes of the teeth; and, of the *morbid* effects produced by teeth deprived of their vitality.

Simple caries is divided by Mr. K. into the *external* and *internal* kinds; and his descriptions of these as well as of their *symptoms* and *causes*, are very good: his treatment, in fewer words than his own, is this:—First of all, he removes all the general and local exciting causes, and then the proximate cause, by extirpating the mortified or inflamed parts of the bony structure of the teeth by surgical operations *properly adapted* to the several stages of the disease: when this has not penetrated more than one third of the bony structure, he cuts away the dead and diseased part with the file and chisel or other instrument, so as to produce a sound and even surface: when it is deeper than one third of the bony structure, yet has not exposed, irritated, or inflamed the nerve of the tooth, the dead and inflamed parts are to be removed and the cavity filled with gold: and, when the caries is deep-seated, it can be cured only by the latter operation; filing or cutting, without stopping, tends to accelerate the disease's course.

Caries is complicated, when it has penetrated as far as the natural cavity of the tooth, and is attended with inflammation and disease of the living membrane: the progress, symptoms, and causes of this morbid state of a tooth are well described. Our author considers tooth-ache as the result of a change in the affected parts from chronic to acute inflammation, determined by internal or external causes altogether foreign to the local disease. It is invariably produced, he says, by some violent irritation of the affected nerve, and not the immediate effect of the disease itself: it may, therefore, be generally palliated or relieved by removal of the causes, or the restoration of the parts to their chronic state, by the assistance of either art or Nature. Mr. K. however, has not chosen to inform his readers of the means by which this palliation or relief is to be obtained; and, hence, for aught we owe to his "observation and experience," the cure of tooth-ache must still remain as much a secret as ever. His treatment of the complicated caries consists in attempting the restoration of perfect health to the tooth's lining membrane and the preservation of its vitality, by subduing the inflammation, or in extracting the tooth. In the first or second stage of this disease, six out of seven teeth generally may be preserved by filing or cutting out the carious parts, and stopping the cavity with gold: when, in the third stage, the lining membrane is acutely inflamed and very painful, or when suppuration has commenced, the morbid tooth should be extracted: and, when any of the temporary teeth are affected with caries in its complicated form, they ought to be removed without delay.

Chap. II. *Morbid Influences of Dead Teeth and Roots.*—The chemical and mechanical actions which remove dead teeth and roots place these under the influence of absorption and putrefaction, and, at the same time, excite inflammation and suppuration in the periosteum and contiguous textures. This process, says Mr. K. produces precisely the same morbid irritation upon the living teeth, their periosteum, gums, sockets, and maxillary bones, as well as upon the general system, as that excited by any other gangrenous, dead or corrosive part of the body, upon the neighbouring parts and the constitution. The truth of these statements, he concludes, will be satisfactorily proved by examination after death. Wherever a dead root or portion of tooth has been left in the mouth during life, the gums and periosteum will exhibit more or less disease, and the traces of inflammation and suppuration; but, in many instances, this morbid state will be distinguishable only by an experienced observer. On dissection, the internal spongy structure of the jaw will evince distinct marks of inflammation and of mortification, extending sometimes to distant parts: occasionally, dark red streaks, approaching very nearly to black, are found: these begin at the points of dead roots and proceed to the cheek-bones, occupying an extent of an inch and upwards. By examining jaw-bones, preserved as anatomical preparations, containing dead roots, the alveoli, says our author, will always be found in a more or less diseased state, and sometimes the caries and mortification are very evident: the parts are generally more or less spongy about the dead roots, and perforated by many holes, through which the matter had been discharged. These remarks indicate close observation and are valuable contributions to pathological anatomy.

Dead roots and teeth give rise to many and intense symptoms: Mr. K's. account of them is good: we give his concluding sentences without alteration.

"Dead teeth and roots," he says, p. 258, "may frequently form the principal cause of many nervous and rheumatic affections of a very alarming nature, and accompanied with much pain; such as, various disorders of the organs of sense, the brain, the face, the ears, the eyes, and other more distant parts, under the forms of tic douloureux, convulsions, epilepsy, hysteria, hypochondriasis, dyspepsy, and mental derangement. Dr. Rush, in his Medical Enquiries upon the *Diseases* of the *Mind*, states a case of madness occasioned by decayed teeth which were not painful: no doubt they were dead teeth. He, also, gives instances of rheumatism in the hip-joint, dyspepsy and epilepsy, cured by removal of painful and dead teeth; and, in confirmation of his opinion, quotes three cases from other eminent physicians.—If those secondary local and constitutional affections, which are frequently mistaken for primary diseases, are treated as such, the symptomatic disorder will generally be much augmented. We cannot, therefore, too much bear in mind, that the idiopathic diseases of the mouth, even when they are in a very advanced state, often occasion the least pain in the parts primarily affected; whereas, the symptomatic disorders produced by them are exceedingly painful and alarming."

When dead roots and teeth are neglected or mismanaged, he adds in another place, they may often produce the most dangerous and distressing primary diseases of the mouth, as inflammation and suppuration, gum-boils, fistulous abscesses, scorbutic excrescences, tumours of the gums, inflammation or suppuration in the periosteum, inflammation and mortification in the alveoli and maxillary bones, and diseases of the maxillary antrum. His surgical remarks on dead roots and decayed teeth are brief and brilliant: those on the "cruel and unnatural practices of Messrs. Hunter and Fox" are exquisitely delicate.

Chap. III. *Absorption of the Gums and Sockets.*—This disease, in Mr. K's mind, is far from being accurately understood, and generally held to be incurable. It usually begins in an inflammation and suppuration of the gums, which gradually extend to the periosteum or alveolar processes; or it commences with inflammation of these parts, which is afterwards communicated to the gums: it very rarely originates in the sockets themselves. In most instances, the inflammation and suppuration are seldom violent, and the absorption so slow as to be scarcely perceptible, and the gums, being gradually destroyed, are attended with absorption of their alveoli and periosteum: in the end, the teeth, losing their support, become loose and successively drop out. Morbid absorption rarely attacks all the alveolar processes at once: most frequently it first invades the grinding teeth. During its course, the crowns, necks, and exposed parts of the roots are often covered with a greenish glutinous substance and adhering tartar, and the spaces between the teeth are filled with a dark brown or greenish tartar. For the most part, also, the gums are a little swelled and inflamed; a thin pus-like discharge flows from between them; and the roots of the teeth yield a very offensive smell. Persons of robust constitution are more liable to this affection than the delicate: its worst forms occur after the age of thirty: and, after forty, it commonly appears in a chronic state. Mr. K. avers that, in most instances, advanced age is unjustly considered to be the cause of this state, and that the disease has a peculiar tendency to attack persons who possess vigorous health and sound teeth; and these, he adds, from the stimulant atmosphere of this country, and the wholesome nourishing diet enjoyed by its inhabitants, are much more frequent in England, than in any other part of the world.

We need not follow Mr. K. through his detail of symptoms which is fair enough: nor is it necessary to transcribe any of his cases; that in which he directed the subserviency of the "humane and skilful," "the excellent," "the sagacious and disinterested," and "the judicious Mr. Lawrence," is particularly choice. *Multum tamen abludit imago.*

Among the remote causes of this disease, our author remarks—a scorbutic and scrofulous habit of the gums, frequent and inordinate use of mercury and narcotic medicines, great irregularities in the position of the teeth, neglect of cleanliness, improper manner of brushing the teeth, smoking and chewing tobacco, abuse of spirituous liquors, operations injudiciously performed, imperfect and unskilful scaling of the teeth, and *certain objectionable* tooth-powders: when the teeth are so much exposed, as to be loosened, they prove a very aggravating cause, and the disease is then in its worst stage.

Mr. K. has succeeded in curing this disease in all its stages by a mode of treatment which consists, in checking the diseased action which has become habitual, and in producing a general healthy disposition in the parts, by removing the actual causes—and then in preventing their recurrence. He attains the first object by extracting the teeth whose periosteum and sockets are incapable of restoration, and by subducing the tartar and glutinous matter from the remaining teeth and their exposed necks : the second end is gained, he says, by removing the remote causes, or, if this cannot be done, by counteracting their effects. There is not any thing extraordinary in his local treatment : the remarks we subjoin merit some consideration.

"Not one of the molars should be permitted to remain that has no antagonist, particularly if it is situated in the upper jaw ; inasmuch as such teeth, being deprived of that necessary stimulus which arises from mastication, their periosteum soon becomes relaxed, and consequently predisposed to disease : besides, the utility of such teeth being lost by want of an opposing surface to act against, they influence the surrounding parts like extraneous bodies. Should there be, for instance, an interval between two opposite teeth when the jaws are closed, Nature uniformly attempts to remedy the evil ; and, for this purpose, the cavity of the alveolus contracts at the base, and drives out the tooth until it meets its opponent, by which the necessary stimulus is restored, and the morbid action cured. In this manner, the loss of one molar tooth produces the destruction of its remaining antagonist. This is effected, however, after a struggle of Nature, of very long duration, which will always involve, in some degree, all the other teeth in a like diseased condition : it is necessary, therefore, to prevent this morbid action by extraction of the tooth, or any molar tooth so situated. Every tooth, moreover, which has lost its vitality, including all stumps, and all such teeth, as from their irregular situation or direction excite a mechanical irritation, provided this irregularity cannot be remedied by filing, or by cutting away the irritating parts, should also be removed."

After these abstractions, Mr. K. advises the bleeding from the sockets to be encouraged by warm water taken into the mouth, between the operations ; and, for several days subsequently, any gentle astringent wash may be employed. In ten or fourteen days more, the inflammation considerably abates, the gums appear more healthy, and the teeth become firm in their sockets. The tartar should now be carefully taken away from the neck or roots of the teeth and the alveolar processes ; after this, the green mucus is to be removed by frictions with tooth-powder and a soft brush ; and, last of all, the re-accumulation of tartar by the use of mechanical and chemical applications :—the rule, says our author, to guide us in the use of these remedies is, that the more the disease has advanced, the more the chemical agency is indicated, and the more carefully is the mechanical power to be applied. After being freed of their tartar, the teeth should be cleaned every morning and after every meal, with an astringent powder, and a brush so hard as not to cause pain. The internal gums are considerably firmer and less irritable than the external, and consequently can bear more pressure and a harder brush : the pressure of the brush is to be applied in the direction *from* the crowns of the teeth towards the roots, so that the mucus which adheres to the roots under the edges of the gums, may be completely detached, and then removed by the friction in a direction towards the grinding surfaces. Mr. K. disapproves of endeavouring to restore the fixity of teeth by means of ligatures. He delays, sometimes for three months, the operations of plugging, filing, or cutting out carious parts, till the irritation caused by them can be endured without injury ; and, it is his opinion, that the introduction of artificial teeth should, if possible, be avoided, except in cases where the necessity is imperious.

CHAP. IV. *Operation of Extracting Teeth and Roots.*—Our author's remarks on the importance of extraction and its remedial effects, contain more truth than novelty, and may be passed with this simple acknowledgement of their merits : his representation of the defects of the "present modes" of extracting teeth and roots, may have some accuracy in them, in as far as they regard the operations, and the operators whom he has had an opportunity of judging : and, in describing the "consequences arising from the present imperfect state of the operation," he aspires to be eloquent :—without pretension to soothsaying, however, we predict that the books of M. Duval will be studied both by dentists and doctors, notwithstanding the severe animadversions of Mr. Koecker. "Were *his* (M. Duval's) system adopted," says our critic, "the art of the dentist would retrograde to *that perfection* in which it flourished between two and three thousand years ago at Athens and Rome, and prodigious improvements might doubtless be expected from so scientific a return to those olden and golden times."

There is nothing remarkable in Mr. K's practical remarks on the operation of extraction: students and young dentists may profit from a perusal of his anatomical and pathological aphorisms. Mr. K had the best opportunity of ascertaining all the effects of the operation upon the patient, under all its circumstances and periods, by taking occasion to extract some of his own teeth : he made the process a matter of particular attention, and thereby acquired personal and experimental information on the point at issue. We transcribe it in his own terms.

"The teeth which I extracted from my own mouth, were seven in number : six upper, and one under, large grinders. They were all very firmly seated in their sockets, and the upper had each of them three, and the under two roots. In the upper teeth, with the exception of one only, the caries had just reached the nerve, and the under was perfectly sound ; so that in six of them no considerable inflammation existed, either in the nerve, or in any of the surrounding parts. The extraction of all the upper teeth was performed with the forceps, and very slowly, that I might be able to feel, and remember distinctly, the peculiar kind of pain attending each part of the operation in all its degrees. When I took hold of the tooth with the instrument, I felt no pain whatever ; in moving it towards the outer side of the mouth, it seemed to me that I could hear the gradual tearing of the periosteum on the inner side, and feel the crushing of it on the opposite side of the roots. This process was accompanied with little or no pain ; but, when the periosteum was perfectly separated and the different nervous cords were being torn off, I felt an acute pain of momentary duration, very similar to what I had experienced the evening before from the tooth-ache for at least an hour ; I believe it was equally great, but certainly not greater, and it was instantly over. The second large grinder, in the left side of the under jaw, was removed towards the posterior part of the mouth ; and, when loosened, was extracted from its socket by the forceps. The lining membrane of the first large grinder, on the right side of the upper jaw, having been previously inflamed, and suppuration having commenced, the pain from extraction was somewhat more acute than what I experienced in the removal of the other teeth, yet it was much more supportable than the tooth-ache."

We come now to a number of sections, having each its own title—as, *of the Performance of the Operation of Extracting Teeth; of the Physical and Mechanical Means to be used in this Operation; of the Operating Chair;*

*of the Forceps; of the Pyramidal Lever; of the Instrument invented by Dr. Physick; of the Key and the Punch; Remarks on Instruments for Extracting Teeth in a Perpendicular Direction; of the Moral Means subservient to the due Performance of the Operation of extracting Teeth; of the Impropriety of applying Force and of using Deception; with concluding Remarks on the Author's Method of extracting Teeth and Roots.* The discussions on those important questions are very good in their way. Mr. K. was reduced to the necessity of inventing an operating chair for himself; and—

" This chair," he states, (p. 340) " is one of the most important improvements which, I hope it will not be deemed presumptuous in me to say, I have made in the apparatus of the profession. It is susceptible of so many changes with great ease and expedition, and without the use of any complicated machinery, that it may be accommodated to the size of any patient, and to every position of the head for any process, and wholly supersedes the necessity of any services from assistants, which are both very uncertain and unsafe."—" The number of my instruments," he adds, (p. 345) " for the extraction of teeth is upwards of eighty, of which more than two-thirds are actually indispensable to do any justice to this important remedy. Indeed, my apparatus, in consequence of its extent, and on account of its great difference from what is generally used, would not only be dangerous in the hands of the ignorant, but its judicious application would require some previous practical instruction even on the part of the scientific dentist."

Chap. V. *Treatment after Extraction, and the Accidents incident to this Operation.*—Mr. K. rejects the use of cold water, vinegar and water, all astringent fluids, brandy and water, and stimulating lotions of every kind, after extraction of a tooth, as being almost invariably productive of pain, inflammation, swelling, sudden mortification of some part of the gums, or other injuries, occasionally of hæmorrhage: he prefers warm water alone, and that of a temperature not to produce any painful sensation in the wounded parts: his only after-treatment is attention to cleanliness of the mouth: when many teeth and roots have been extracted, and the parts had been previously diseased, he endeavours to promote absorption by slightly stimulant or astringent lotions: but these must always be warm, and of very moderate strength. When a tooth breaks during extraction, he proceeds immediately to remove the roots; and, on considerable portions of the sockets and jaw-bone being broken, he smooths the irregular corners, and applies a stimulant lotion, mixed with something mucilaginous. He condemns, as " dangerous and pernicious," the practice of replacing a sound tooth extracted by mistake. In all cases of hæmorrhage after extraction of a tooth, he denounces as mischievous all tonic aad stimulant or acid lotions, and caustics of every kind, especially the actual cautery, and advises, in their stead, the use of water as warm as can be comfortably borne in the mouth; but his principal dependence is on the application of mechanical pressure. His three histories of hæmorrhage are very deserving of attention.

Chap. VI. *On Stopping Carious Teeth.*—We cannot afford space to introduce our author's practical remarks on stopping the teeth, or his criticisms on the present imperfect mode of performing this operation: those at the expense of Mr. Fox are very severe as usual. Neither is it requisite that we should detain our readers with his estimate of the instruments in use for the process of stopping; this, therefore, with his descriptions of the materials improper for such a purpose, and of the methods of employing them, which he deems injurious, we pass over; and, in doing this, will arrive at his own method of plugging the teeth. "On looking over my cases of instruments, I find," he says, (p. 403) "that, for this operation alone, more than one hundred and seventy separate implements are ready for use; not to add, that I am always provided with some particular mechanical tools with which I can, at any time, modify certain instruments, with a view to adapt them for uncommon cases with very little delay."

Mr. K. declares that gold is the only material, the durability of which can be depended upon for properly plugging the teeth: he uses the metal in its purest state, and generally keeps about six different degrees of thickness of the gold leaves, for the purpose of being prepared to meet every diversity of circumstance. All carious teeth, in which the disease has not "completed its work of destruction," nor has advanced to such extent as to be incurable by the combined efforts of art and nature, indicate the necessity for this operation: our author's general rules relating to this question, will be perused with advantage. To render the process certain of success, it is requisite that the morbid teeth be perfectly free from all symptomatic inflammation of the lining membrane or bony structure: general fever, however slight, is a sufficient cause for delaying the operation: it should never be attempted, indeed, except when the individual enjoys good health: every local exciting cause of inflammation should also be entirely removed.

Stopping a tooth is divided by Mr. K. into two parts—a curative, and a preventive treatment: the first consists in the perfect extirpation of the carious, or, as he calls it, the bony abscess; the second, in the proper stopping of the cavity, on which the permanency of the cure depends, and a relapse is prevented: by thus filling up the cavity, three great objects are attained—security against any accumulation of corrosive matter in it; protection of the lining membrane; and restoration, in a great measure, of the health and strength of its bony structure. Great care should be taken to cut away the whole unsound portion of the tooth; not merely what is brown or darker than the rest, but every particle that is not white and evidently undiseased: if one speck of caries remains, a cure cannot be accomplished. When the lining membrane is covered only with dead or putrid structure, this should be perfectly removed, and when the nerve is laid bare and bleeds, the hæmorrhage must be allayed before the plugging can succeed.

"The final consideration," says the author, (p. 416) "in the management of this part of the operation is, to take great care to give the cavity a proper form in order to retain the metal. For this purpose, it should be round or oval, entirely smooth, and free from ragged edges," (*and, he should have added, wider at the bottom than the orifice;*) "and, before the metal is placed in the tooth, the cavity should be carefully washed out with locks of cotton" (*a soft feather, a hair pencil, or piece of sponge fixed to a proper handle is preferable*) "dipped in warm water, and afterwards completely dried by the same means. In stopping the tooth, the metal should be firmly pressed into the cavity, and rendered as compact as if it were solid metal, so that nothing could by possibility penetrate through it. The redundant metal is then to be cut away, and the plug perfectly smoothed and polished by some burnishing instrument."—"When the caries is in that side which is next the adjoining tooth, a division must be made with a file between the teeth, in an oblique direction towards the neck of the tooth, so as to permit extirpation of the caries and filling of the cavity."

☞ *Continued at p.* 213 *et seq. of this Number.*

(*Concluded from page* 144.)

Chap. VII. *Treatment of the Lining Membrane or Nerve, when exposed, previously to Stopping.* Mr. K. by way of introduction to this, his final chapter, shews conclusively that the common method of managing exposure of these parts, is "both cruel and contrary to reason;" and then, having done this, proceeds to expound the "proper and judicious treatment," which he "has practised for upwards of thirteen years, with much satisfaction and success." It is "the only one calculated to ensure success," and is this—1st. After removing every cause of symptomatic inflammation, to arrest the caries, and consequently to prevent irritation of the internal membrane; 2d. To suppress the hæmorrhage, and to cure the wound of the membrane, if it be wounded; and 3d. To protect the membrane artificially against the action of all foreign agents.

The first of these objects is to be obtained by extirpating all the carious particles, and, if the lining membrane has not been injured, by immediately plugging the cavity with gold. When the membrane has been wounded and a hæmorrhage ensues, he attempts to stop the bleeding with weak acids and styptics; and, on these failing, he resorts to the actual cautery for the purpose of effecting an artificial contraction of the wound: he uses a red hot wire for this purpose, and performs the operation very adroitly and without any loss of time. When the hæmorrhage has been arrested in this way, and a cicatrix formed, he washes the cavity with warm water, so as to make it perfectly clean; and after waiting till Nature has ultimately healed the wound, completes the process by filling up the cavity in the tooth with metal. This is done by first laying a small plate of very thin lead-leaf upon the exposed nerve and surrounding bony parts, and then plugging the aperture accurately with gold. He covers the nerve with lead, because he "believes this metal has a *cooling and anti-inflammatory effect upon the irritated nerve*, at least, it possesses these qualities in a greater degree than gold." Subsequently to the operation, the patient should guard against imprudent exposure to the damp and cold air, and to every sudden change of temperature, and the teeth must be kept clean by the use of some suitable dentrifice, good brushes, and warm water. When there is a tendency in the constitution to inflammatory excitement, the gums ought to be often bathed with tincture of myrrh and warm water, in equal parts: if the tooth is painful, the gums must be scarified and the bleeding promoted by warm fomentations: and, when the blood ceases to flow, the myrrhine lotion, with a little honey, is to be continued. Mr. K. has seldom found benefit from the application of leeches to the gums: we have. In many instances, the pain ceases with the operation; but, sometimes, the tooth becomes occasionally painful during several months: as long, however, as it possesses vitality, there are hopes of a cure. When the tooth grows loose and has a pale colour, and the gums are red, swollen, suppurating and painful, with ulcers and fistulous openings, the nerve is destroyed, and the tooth should be forthwith extracted. If, after three months, the tooth retains a natural appearance, and is free from pain without being insensible or loose, the cure may be pronounced as complete: five out of six teeth, our author avers, can be preserved alive by such treatment.

We do not, in conclusion, expect Mr. Koecker will be over well satisfied with our remarks on his book, but we trust that our readers will. Throughout the article, we have essayed to secure approbation for the best parts of the volume, many of which appear to be original; and, we are sure, likewise, that no injustice has been done to those which are his only in common with other writers on dental surgery. Our chief objections relate to the excess of his severity; and to the assiduity wherewith he has picked out, for animadversion, whatever weak parts could be detected in the writings of others. The manner also in which his strictures have been made on Hunter, Fox and Duvâl, is objectionable: in regard to the two former, he should have been pleased to remember, that bare justice at the very least, was due to the illustrious dead; for both of these surgeons were excellent philosophers, and each of them contributed infinitely more to the advancement of dental physiology and pathology, than the small number of their defects will conduce to retard the progress of these intricate branches of knowledge. Mr. Fox, for instance, directs that, "in stopping a tooth, a piece of gold or tin-foil leaf is to be introduced, and carefully and firmly pressed in, so as completely to fill up the cavity;" and, at the end of the section, mentions merely, but does not at all recommend, a fusible metal, pointed out to him by some chemists, as being applicable to the same purpose: it was, therefore, uncharitable to select this as a subject of severe sarcasm, and at the same time pass over in silence the very practice our author himself employs. A comparison, moreover, of M. Duval's doctrines with those of Mr. K. would elicit something by which the latter might be edified. In fine, by condensing his volume into one-third of its present size, and by unveilng his practical rules of all sort of mystery, Mr. K. will transform it into a useful work, and obtain for himself the dignity of an honourable and zealous cultivator of his profession.

We have reason to know that Mr. Koecker is a very excellect practical dentist; and that the zeal and ability with which he manages every operation he undertakes, are truly praise-worthy. These qualifications will, we trust, ensure him patronage and success in this metropolis; but we would have proved ourselves his greatest enemies, if we had failed to point out to him the objectionable parts of his writings.

